# Auditory Verbal Hallucinations in Persons With and Without a Need for Care

**DOI:** 10.1093/schbul/sbu005

**Published:** 2014-06-13

**Authors:** Louise C. Johns, Kristiina Kompus, Melissa Connell, Clara Humpston, Tania M. Lincoln, Eleanor Longden, Antonio Preti, Ben Alderson-Day, Johanna C. Badcock, Matteo Cella, Charles Fernyhough, Simon McCarthy-Jones, Emmanuelle Peters, Andrea Raballo, James Scott, Sara Siddi, Iris E. Sommer, Frank Larøi

**Affiliations:** ^1^King’s College London, Institute of Psychiatry, Department of Psychology, London, UK;; ^2^South London and Maudsley NHS Foundation Trust, London, UK;; ^3^Department of Biological and Medical Psychology, University of Bergen, Bergen, Norway;; ^4^The University of Queensland Centre for Clinical Research, Metro North Mental Health, Royal Brisbane and Women’s Hospital, Brisbane, Australia;; ^5^King’s College London, Institute of Psychiatry, Department of Psychosis Studies, London, UK;; ^6^Department of Psychology, Universität Hamburg, Hamburg, Germany;; ^7^Institute of Psychological Sciences, University of Leeds, Leeds, UK;; ^8^Department of Education, Psychology, Philosophy, University of Cagliari, Cagliari, Italy;; ^9^Department of Psychology, Durham University, Durham, UK;; ^10^School of Psychology, University of Western Australia, Crawley, Australia;; ^11^National Institute for Health Research (NIHR), Biomedical Research Centre for Mental Health at South London and Maudsley, NHS Foundation Trust, London, UK;; ^12^ARC Centre of Excellence in Cognition and Its Disorders, Department of Cognitive Science, Macquarie University, Sydney, Australia;; ^13^Department of Mental Health and Pathological Addiction, AUSL Reggio Emilia, Reggio Emilia, Italy;; ^14^Psychiatry Department, University of Utrecht, Utrecht, The Netherlands;; ^15^Department of Psychology, University of Liège, Liège, Belgium

**Keywords:** nonclinical, need for care, psychosis, prevalence

## Abstract

Auditory verbal hallucinations (AVH) are complex experiences that occur in the context of various clinical disorders. AVH also occur in individuals from the general population who have no identifiable psychiatric or neurological diagnoses. This article reviews research on AVH in nonclinical individuals and provides a cross-disciplinary view of the clinical relevance of these experiences in defining the risk of mental illness and need for care. Prevalence rates of AVH vary according to measurement tool and indicate a continuum of experience in the general population. Cross-sectional comparisons of individuals with AVH with and without need for care reveal similarities in phenomenology and some underlying mechanisms but also highlight key differences in emotional valence of AVH, appraisals, and behavioral response. Longitudinal studies suggest that AVH are an antecedent of clinical disorders when combined with negative emotional states, specific cognitive difficulties and poor coping, plus family history of psychosis, and environmental exposures such as childhood adversity. However, their predictive value for specific psychiatric disorders is not entirely clear. The theoretical and clinical implications of the reviewed findings are discussed, together with directions for future research.

## Introduction

Auditory hallucinatory phenomena occur on a spectrum ranging from auditory imagery and intrusive and vivid thoughts to fully developed hallucinations of hearing sounds and voices. Although traditionally associated with psychiatric and neurological diagnoses, hallucinations may also be present in healthy individuals without need for care. It has been observed that individuals with AVH vary widely in their need for care, and clinical status may change over a person’s lifetime. Understanding the factors that are relevant in leading to or protecting from need for care can inform clinical interventions. This article brings together research findings on auditory verbal hallucinations (AVH) in the general population and considers the clinical relevance of these experiences. We cover the different methodological approaches that have been adopted to elucidate the factors related to the process of “transition” to a need for care, including longitudinal epidemiological studies, as well as comparison of AVH present in persons with and without a need for care. In this review, we combine the various, often isolated, research streams on multiple aspects of AVH into a cross-disciplinary overview, which documents areas of emerging consensus as well as highlighting contentious and underresearched domains.

The article was initially prepared for the Second Meeting of the International Consortium on Hallucination Research (Durham, UK, September 2013). Beforehand, the authors created a list of topics considered important and/or neglected in the area of AVH in persons with and without a need for care. Then, the authors worked in small groups to expand on these areas, by analyzing the literature and drawing on their own expertise and research findings, in order to extract the key components in understanding the continuum of AVH experience and risk for clinical disorder.

## What Is the Prevalence Rate of AVH in the General Population?

Only a few studies have specifically examined the prevalence of AVH in the general population. The reported prevalence varies widely: in a historical overview of 17 studies from the late 19th to early 21st century,^1^ the rates of AVH ranged from 0.6% to 84% (median: 13.2%). Linscott and van Os^2^ retrieved 56 reports containing data on rates of psychotic symptoms in adult community samples and report a median lifetime prevalence rate of 4.1% for hallucinations (all hallucination types grouped together). In the first cross-national (52 countries) study,^3^ an age- and gender-adjusted estimate of 5.8% for hallucinations (all types grouped together) was reported, but with highly varying prevalence rates across countries (from 0.8% in Vietnam to 31.4% in Nepal). The rates appear higher in children and adolescents: Kelleher et al,^4^ in their meta-analysis, found a prevalence of 14.8% in children and adolescents (age range 9–18 years) specifically for AVH. Moreover, there were no clear differences between rates for children versus adolescents (13.8% and 15.7%, respectively) (also see Jardri et al^5^).

A limit with basing prevalence rates on meta-analyses is the methodological heterogeneity in how AVH are assessed, the timeframe used, and characteristics of the population, resulting in large variations in prevalence rates (cf. Beaven *et al* ^1^). Also, important nuances are lost, such as the nature and frequency of the AVH. For example, in Johns et al,^6^ 4.2% of the general population surveyed answered affirmatively to a general hallucination item (“Over the past year, have there been times when you heard or saw things that other people could not”), whereas only 0.7% endorsed a more specific AVH item (“Did you at any time hear voices saying quite a few words or sentences when there was no one around that might account for it?”).

## Is There a Continuum of Hallucinations and Psychosis?

A dimensional view posits that (1) AVH and other psychotic experiences lie on a continuum with normal experience^7^ and (2) psychosis exists in the population as a continuous phenotype.^7^ Such a continuum model is helpful for understanding AVH in terms of normal cognitive processes and facilitating research into etiological factors and clinical trajectories. Two types of continua can be distinguished, both within and across individuals^9^: (1) A continuum of experience, whereby different experiences (daydreams, intrusive and vivid thoughts) lie on a common continuum with AVH; (2) a continuum of risk, in which people differ in (a) their proneness to experience AVH and (b) their risk of developing problematic AVH with need for care. This section considers the continuum of AVH experience across individuals (phenomenological continuity), including the continuum of risk for psychosis (structural continuity), and reviews evidence for and against putative continua. Although there is robust evidence for a continuum of psychotic experiences, with a distribution in the general population, there is less evidence that this represents a single underlying continuum of risk for psychosis.^10,11^

### Evidence for a Continuum

Phenomenological continuity is indicated by studies showing that more people experience AVH and other psychotic experiences than those individuals who receive psychiatric diagnoses,^12^ with a range of reported hallucination prevalence rates in nonclinical samples. Further, in these population samples, hallucinations are correlated with delusions, just as they are in psychotic disorders.^13^

Evidence for structural continuity (a single group in the population with quantitative variation in phenotype expression) comes from similar associations between key risk factors and both psychotic experiences and psychotic disorder, suggesting etiological continuity between them.^14^ These risk factors include younger age, ethnic minority status, lower education, alcohol and drug use, stressful or traumatic events, urbanicity, and family history of psychotic disorders.^6,15^ Other evidence for etiological continuity comes from direct comparisons of individuals with AVH with and without need for care, which reveal partly similar neurocognitive processes and brain regions underlying AVH in both. This suggests common cognitive mechanisms across the continuum of AVH experiences irrespective of clinical status, but with some cognitive difficulties increasing in severity along the continuum of risk for AVH with a need for care.

A fully continuous relationship between psychotic symptoms and disorder can be distinguished from a quasi-continuous/continuum-threshold model.^13,16^ The latter is more consistent with the observed skewed distribution of AVH in the population, qualitative differences in these experiences along the continuum, and the contribution of various risk factors in making “transitions” from nonclinical to clinical states. Findings from studies comparing the AVH reported by individuals with and without need for care indicate that in addition to similarities, there are also specific differences in the experience, and possibly the underlying mechanisms, of AVH across the continuum, some of which might contribute to clinical status.

### Evidence Against a Single Continuum

Factors of individual difference,^16^ general psychological distress,^17^ and psychosis proneness^12^ could either determine where a person lies on a continuum of AVH or reflect different interacting continua (which give rise to an apparent single continuum). There is emerging evidence that a latent categorical structure of the population underlies the observed continuum of psychosis experience,^2^ with 1 group who are liable to psychosis and another group who are not. In the former, AVH are associated with other cognitive and emotional difficulties and a greater likelihood of need for care, while in the second group, AVH have reduced morbidity and possibly different etiology.^18^ This could partly explain why 2 people with the same level of AVH may differ in their clinical outcome. So, although AVH and other psychotic experiences seem to be continuous and distributed across the general population, the risk for developing psychosis might actually be discontinuous rather than truly continuous in the population. Further research is needed to understand these different continua and any underlying factors that could potentially serve as biomarkers.

## What Are the Similarities and Differences in the AVH Reported by Individuals With and Without Need for Care? (see figure 1)

### Phenomenology

AVH in individuals with and without need for care are broadly similar as perceptual phenomena and in terms of topographical features such as localization (internal or external), loudness, and number of voices.^19,20^ Regardless of need for care, hallucinators tend to personify their voices (ie, attribute their voices to a real person or entity)^19^ and seem to share similar underlying brain activity.^21^ Differences lie largely in the frequency and duration with which voices are experienced and age of onset, with the nonclinical group starting to have AVH at a younger age, often in childhood.^19^ The most significant differentiating factor, however, is the degree of negative voice content, with patients reporting a preponderance of negative voices, while AVH in individuals without need for care are mostly neutral or pleasant in content.^19,20,22^ This suggests that negative content is crucial in determining increased distress and need for care.^23,24^ However, many patients also report some positive voice content,^25^ and it is important to examine the balance of negative and positive voices a person hears.

**Fig. 1.**
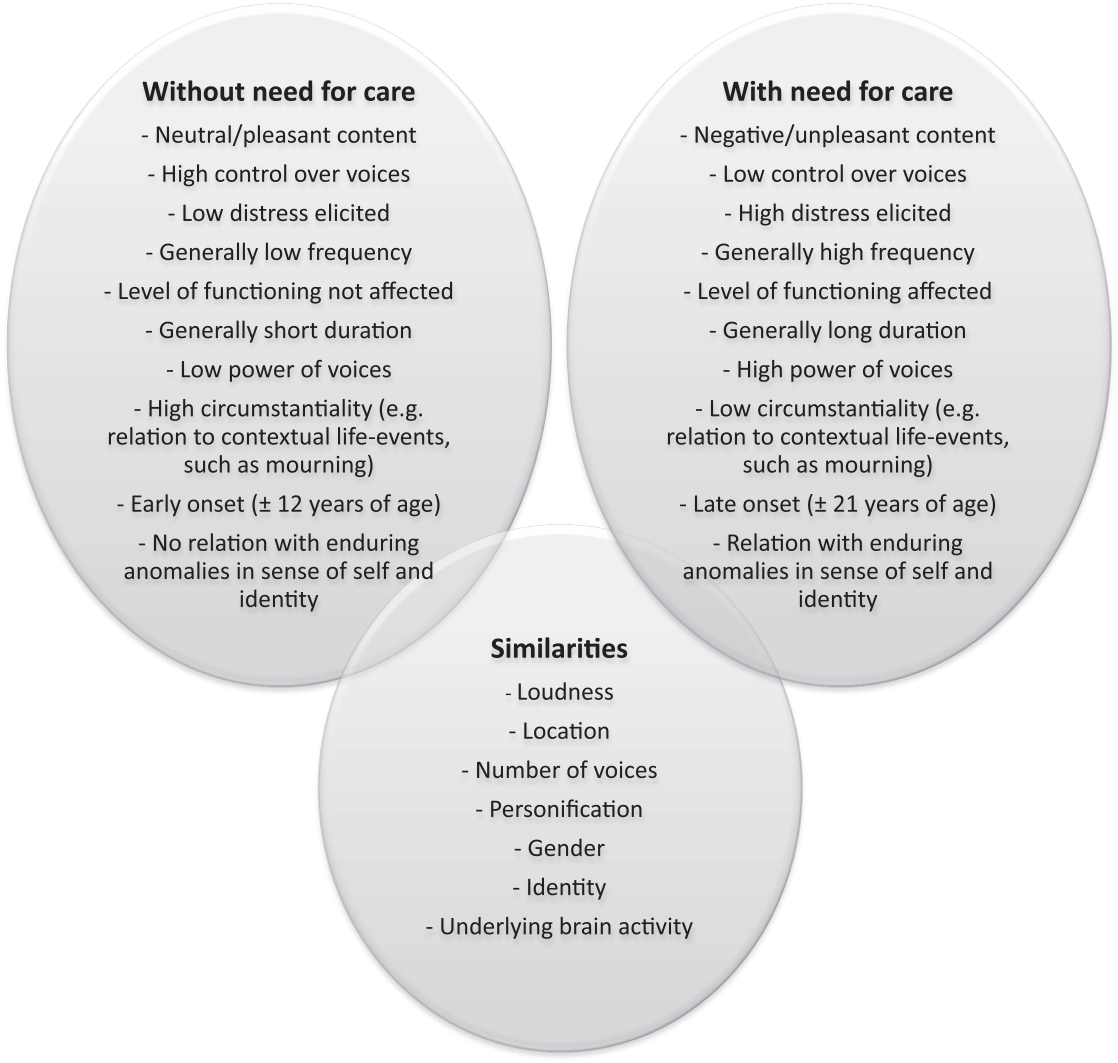
Principal differences and similarities between auditory verbal hallucinations experienced by persons with a need for care and those without a need for care.

### Cognition

Regardless of need for care, individuals with AVH have difficulty on tasks measuring cognitive control functions, such as controlling the direction of attention in the face of distracting information and the active suppression of intrusions. A meta-analysis^26^ of 9 studies on source monitoring in individuals with AVH without need for care found a significant, moderate-to-large effect, which did not differ from findings in individuals with AVH with need for care. However, given the relatively small number of studies, further replications are desirable before concluding with certainty that individuals with AVH have a specific, similar-sized source monitoring difficulty independent of their need for care. Due to the range of dysfunctional control components (such as intrusive cognitions, source monitoring, and inhibitory control) in cognitive models of AVH,^27^ studies of cognitive control functions dominate the literature on cognitive correlates of AVH. Future studies should concentrate on detailing the pattern of affected and intact subcomponents of executive functioning in persons with AVH (cf. Waters *et al* ^28^).

Memory has also been investigated in hallucinators without need for care. No outstanding deficit appears in control-demanding episodic long-term and short-term tasks nor in binding memories to a specific context (see Badcock et al,^29^ Chhabra et al,^30^ and McKague et al^31^). Thus, it appears that lapses in cognitive control in these individuals are not coupled to wider difficulties in memory processing. This represents a potential discontinuity between the cognitive profiles of individuals with AVH with and without need for care^27^ and could furthermore be a future target for cognitive training interventions.

Given the contribution of low-level sensory and perceptual processing to AVH in individuals with need for care,^32^ more detailed examination of these influences would be valuable in persons with AVH without need for care. For example, hallucinating individuals without need for care and nonhallucinating controls process various acoustic dimensions of voices similarly, whereas hallucinators with need for care rely less on certain acoustic features.^33,34^ On the other hand, increased tone detection threshold has also been shown in individuals with AVH without need for care,^35^ suggesting similarity with clinical groups in very basic auditory functions. Furthermore, cognitive dysfunction is a frequent symptom of patients with psychotic disorders, especially those with a diagnosis of schizophrenia. People with AVH without need for care, however, tend to have cognitive functioning within the normal limits.^36^ It is possible that intact cognitive functioning is a major protective factor for people with AVH who do not develop need for care. More research on this topic is needed.

### Neurobiology

Structural neuroimaging in individuals with AVH and a need for care has shown that gray matter loss in superior temporal regions^37^ as well as the insula^38^ is associated with hallucination severity. It is not yet known whether the same applies to people with AVH without a need for care. A structural connectivity study has suggested similar alterations in the microstructure of the arcuate fasciculus in hallucinating individuals with and without a need for care^39^ compared with nonhallucinating controls. Functional neuroimaging suggests that the neural correlates of experiencing AVH are the same in individuals irrespective of need for care. Thus, neuroimaging studies on the “state” (vs trait) of hallucinations in people with and without need for care have not observed significant differences in activation of the brain regions involved.^40^ However, some possible markers for transition to need for care have been found, such as elevated striatal dopamine capacity, which appears to be specific for predicting psychosis but is not associated with the presence of AVH per se.^41^ Similarly, Diederen et al^42^ suggest that decreased functional lateralization, a mechanism proposed as important in the development of AVH,^43^ is specific to psychosis because they found no evidence for functional lateralization in individuals with AVH without need for care. However, within schizophrenia patients, there appears to be a continuous relationship between the severity of AVH and degree of functional lateralization.^43,44^

### Life Events

Robust associations have been found between traumatic life events and AVH in both those with need for care^45–47^ and without need for care.^48^ Large-scale epidemiological studies of the general population have observed the same association when controlling for a range of confounds.^49^ More recent work has examined whether the type of trauma experienced predicts need for care. Daalman et al^48^ found no differences between 100 psychiatric patients with AVH and 127 individuals with AVH without a need for care in prevalence of specific types of abuse: both groups were more likely to have experienced sexual as well as emotional abuse than were nonhallucinating control participants. Goldstone et al^50^ modeled hallucination proneness among 100 patients with psychotic disorders and 133 students. In the student sample, emotional trauma in childhood, combined with proximal life stressors, was the strongest predictor of proneness to AVH, while sexual abuse was the strongest predictor in the clinical group.

Although there is no clear evidence of trauma type differentiating need for care and non-need for care groups with frequent voices, traumatic stressors may be of lower impact in those without a need for care, and their psychological sequelae may be less persistent. Thus, while sexual and emotional trauma may initiate hallucination onset per se, it may be the psychological impact of the trauma that encourages the development (and maintenance) of clinically significant AVH by negatively influencing beliefs about voices, which in turn predicts the levels of distress and impairment experienced.^22^ McCarthy-Jones^51^ has proposed that 2 specific posttrauma factors may promote the development of negative AVH. The first is the degree of shame and self-blame the person feels in relation to the traumatic event, and the second is the degree of social or emotional isolation following the trauma. McCarthy-Jones^51^ argues, following Romme et al,^52^ that it is these emotions and the failure for them to be expressed, which form the basis for the negative content of AVHs. However, this hypothesis remains to be tested. The high rates of trauma exposure in people with AVH both with and without need for care support the need for continued research into relevant developmental events and additive vulnerabilities to understanding the pathways to the distress and disruption that necessitates clinical care.

### Appraisals, Coping, and Relationships

The cognitive model of voices proposes that the beliefs people hold about their voices^53,54^ and their social schemata^55,56^ mediate the relationship between the voice experience and behavioral and affective response. There is accumulating evidence that appraisals about identity,^32^ intent and power,^57^ and the nature of the relationship with the personified voice^58–60^ are more important determinants of distress and disruption than voice activity per se.

A number of studies have compared voice appraisals, and the relationship between the voice and the individual, in people with AVH with and without a need for care. Individuals without a need for care report higher perceived control over their voices than do people with a need for care.^22^ They also display less symptomatic coping^61^ (ie, going along with the content of voices), engage in fewer safety behaviors in relation to their anomalous experiences,^62^ and score lower on maladaptive response styles in response to experimentally induced anomalous experiences.^63^ Voice hearers with a need for care are more likely to attribute their voices to real people or agencies, as opposed to spiritual or religious sources,^19^ and generally have more “paranoid” appraisals both of their own experiences^64,65^ and of experimentally induced anomalous experiences.^63^ They are more likely to appraise their voices as malevolent, omnipotent, intrusive, dominant, and coercive and, consequently, are more likely to resist them and keep their distance.^22,66,67^ Individuals with a need for care also display more cognitive biases^68^ and negative metacognitive beliefs about thoughts.^20^ It is possible, however, that these differences in appraisal are secondary to differences in emotional content, an issue that deserves further attention.

### Strengths and Limitations of This Approach

Comparing AVH experienced by groups of people with and without a need for care suggests some factors that may explain why some hallucinators develop a clinical status and others do not. However, if the proposed latent categorical structure of the population is correct in relation to psychosis, then studies comparing hallucinating individuals with and without psychosis/need for care might well be comparing participants drawn (in varying proportions) from 2 qualitatively distinct groups, which will confound their findings. Furthermore, these studies might not be comparing like with like in terms of the phenomenology of the AVH or the severity of other symptoms, such as delusions or cognitive dysfunction, across the 2 groups.^41^ Cross-sectional comparisons are limited in answering questions about the continuum of risk for developing AVH with need for care, which are best addressed with epidemiological and longitudinal studies.

## What Can Nonclinical Hallucinations Say About Risk for Psychosis and Need for Care?

### Clinical Outcomes of Those Who Hallucinate

There are various outcomes of those who experience AVH in early life in terms of continued experience, mental health status, and functioning. The hallucinations may cease or continue with no negative impact; indeed, the most common outcome of hallucinatory and other psychotic-like experiences in childhood is discontinuation of these experiences^69,70^ (see also Jardri et al^5^). For instance, Bartels-Velthuis et al^71^ report that as many as 76% of children who reported hearing voices at 7 and 8 years of age stopped hearing voices by age 12–13. However, for others, AVH persist into adolescence and adulthood and can develop in some people to psychotic disorder or other diagnosable mental health problems. Longitudinal cohort studies have shown that hallucinations and other psychotic symptoms in children and adolescents are associated with an increased risk of later diagnosis of mental illness, but results diverge on whether adolescent AVH specifically increase the future risk of psychotic disorders.^72–74^ It is still not wholly understood why some individuals with AVH develop particular adverse mental health outcomes although a number of specific factors have been identified, which converge with those identified by cross-sectional studies.

### Psychological Mechanisms Mediating Transition to Psychosis

The literature suggests 3 key psychological factors that seem to influence risk for developing a psychotic disorder in those with nonclinical AVH: Cognitive biases, negative affect, and coping style. These interdependent processes may synergistically increase psychosis risk by fuelling the impetus for delusion formation and elevating distress associated with hallucinations.^24,75^

#### Cognitive Biases.

 Various idiosyncratic cognitive processes are implicated in transitioning to AVH with need for care, but the mechanisms are sometimes difficult to test directly. These processes include a jumping to conclusions (JTC) reasoning bias, hypervigilance to threat-related stimuli, externalizing and personalizing attributional biases, contextual information integration difficulties, source monitoring errors, and poor Theory of Mind skills.^76,77^ It remains unclear how much these processes contribute to the development of clinical status rather than the occurrence of AVH per se. Top-down decision-making and thinking biases, such as intentionalizing and JTC, seem to be involved in the transition to clinical psychosis,^68^ whereas bottom-up cognitive processes are important for the formation of AVH across the continuum. The influence of top-down cognitive processes on clinical transitions is supported by findings from the longitudinal Netherlands Mental Health Survey and Incidence Study (NEMESIS), in which onset of delusional ideation at 1-year follow-up increased the risk of psychosis at 3-year follow-up in those with hallucinations at baseline.^24^

#### Affect.

 Negative emotional states play a role in both the onset and maintenance of psychotic disorder. The NEMESIS study found that the individuals reporting hallucinations at baseline who developed depressed mood a year later were at increased risk of developing a psychotic disorder 3 years later.^78,79^ Anxiety levels have also been found to be predictive of higher levels of distress in those experiencing AVH,^80^ which may lead to need for care. Negative affect seems to influence transition to clinical states in various ways: Associated negative thought content can lead to more negative voices^51,81^; emotional states may exacerbate relevant cognitive biases^82^; and depressed and anxious mood can reduce effective coping.

#### Integrating These Psychological Factors.

Appraisals of hallucinations are given central importance in cognitive models of psychosis development.^75^ Cognitive biases impact on appraisals of voices, whereby unusual and confusing experiences that seem caused by an external agency are appraised as such. External and personalizing appraisals, particularly those that are threatening and with lower perceived control, are likely to produce feelings of distress and unhelpful reactions of either preoccupation or avoidance, which may ultimately result in need for care. Appraisals of voices influence coping action^83^ and voices construed as benign have been found to be associated with a greater range of coping strategies.^84^ Conversely, Escher et al^70^ found that adolescents feeling overwhelmed by the experience of voices at baseline used more defensive coping responses and were more likely to develop depression over a 3-year follow-up period. As mentioned, this negative mood may also impact on appraisal process, fuelling the process of development of further psychotic symptoms and disorder. In addition, distal and proximal environmental factors, such as adverse life events, stress, and isolation, have an impact on these psychological processes. For example, Bartels-Velthuis et al^69^ found that exposure to childhood adversity increases the intrusiveness of the hallucinatory experience, together with distress and external locus of control, all of which may increase the risk of secondary delusional ideation.

In summary, there are individual differences in the proneness to experiencing AVH, possibly related to differences in auditory function, cognitive control, self-monitoring, and dissociative tendencies. The association between trauma and AVH suggests that something about the experience of trauma influences the cognitive and emotional processes that give rise to AVH, and there may be more than 1 etiological process.^85^ It is not clear why AVH persist in some individuals or the prognostic significance of this. The onset of AVH tends to be younger in individuals without a need for care, suggesting that persisting AVH are not always clinically relevant. On the other hand, longitudinal studies have found that persistence of AVH into adolescence is associated with negative clinical outcomes.^76^ Studies indicate that, in addition to psychotic disorders, AVH of similar phenomenology are associated with a number of other psychiatric diagnoses, including bipolar, borderline, and dissociative disorders.^86^ We have yet to elucidate the factors that determine these different clinical trajectories. The biased focus on psychosis and schizophrenia as a risk for individuals reporting AVH draws attention away from other disorders that are associated with AVH.

## Discussion

This review raises a number of questions and directions for future research, which are considered below.

### Are There Multiple Types of AVH Without Need for Care?

There are differences among nonclinical hallucinators, reflecting either different points on a single continuum of AVH or separate subtypes.^9,87^ Our label “individuals with AVH without need for care” may thus be further divided into (1) “Hallucination-prone” individuals, who experience brief AVH infrequently, usually under specific conditions (eg, sleep deprivation, mourning); there are few other subclinical symptoms, and these AVH do not affect the person’s functioning; (2) “Nonclinical voice hearers,” who experience more frequent AVH of longer duration. These AVH are often associated with other subclinical psychotic and mood symptoms. There may be a family history of psychiatric illness, and the degree of need for care may vary (see below). These 2 groups are usually based on different assessment strategies: The former is determined using hallucination-proneness measures (eg, the Launay-Slade Hallucination Scale, see supplementary appendix), whereas the latter group is often assessed with interview schedules (as in Sommer *et al* ^88^). This distinction is important when understanding the literature and designing future studies, as findings will be affected the way participants are assessed and grouped in terms of their AVH. For instance, the different results observed in the Daalman et al^48^ and Goldstone et al^50^ studies examining the role of trauma in AVH may be related to the type of participants recruited: Daalman et al^48^ included a nonclinical group who heard frequent voices, whereas Goldstone et al^50^ assessed hallucination-prone students.

### What Is the Predictive Value of AVH?

A crucial question is whether experiencing AVH predicts the development of clinical states and/or future need for care. AVH in children mostly cease spontaneously before adolescence, but persistence during and beyond adolescence is associated with greater risk of developing various clinical disorders.^76^ The psychosis proneness-persistence-impairment model^12^ attempts to explain this trajectory for psychosis, whereby psychotic experiences that become more numerous and persistent over time (due to an interaction between psychological factors, environmental exposures, and genetic risk) increase the probability of onset of clinical psychosis. The presence of AVH might give rise to secondary delusional ideation (to explain AVH), resulting in the emergence of a combined “hallucinatory-delusional state,” which may then elicit negative emotions and maladaptive coping, plus other symptoms, leading to functional impairment and a diagnosable psychotic disorder.^69^ The need to explain the often unusual nature of AVH seems a logical necessity for human behavior and might be more likely for AVH that persist rather than transient experiences or when the person experiences AVH as an adolescent or adult rather than as a child. Furthermore, this need for explanation is influenced by cognitive biases,^75^ which may be related to a separate risk for psychosis that interacts with the presence of AVH.

In adult samples, some individuals with AVH “without a need for care” may experience other difficulties for which they do develop a need for care although not requiring specialized psychiatric services. Their AVH might be a reaction to life stress or change, formulated as a symptom of distress or a type of coping mechanism.^76^ The sample of nonclinical voice hearers described by Sommer et al^88^ had additional subclinical symptoms (delusional ideation, schizotypy) and slightly reduced global functioning. In a general population sample (excluding those with clinical psychosis),^89^ it was found that hallucinations in the past year were associated with seeing a family doctor for emotional problems and with counseling/therapy although were not an independent predictor of family doctor attendance after controlling for other help-seeking correlates.

### What Are the Clinical Implications?

If transitions to clinical AVH and distress are influenced by cognitive processes, appraisals, and coping styles, then psychological interventions can target these factors (see Thomas et al^90^), both to prevent or delay transition to clinical states and to promote recovery in those with AVH and need for care. Cognitive behavior therapy aims to reduce distress and empower the individual by modifying threatening appraisals and building up a normalizing view of voices.^91^ Similarly, acceptance and commitment therapy aims to reduce experiential avoidance of AVH and foster adaptive coping.^92^ Compassion-focused therapies for improving self-esteem and reducing shame associated with trauma can reduce the negative content of AVH.^93^ Emotion regulation strategies may be helpful if AVH are triggered by intense distress, together with formulating the emotional conflict.^94^ Reasoning training^95^ and metacognitive training^96^ are promising interventions for reducing cognitive biases such as JTC, and cognitive remediation can address various cognitive control and executive functions underlying AVH.^97^

In addition to psychological factors, we can look to the cultural context (see Luhrmann et al^98^) and how accepted AVH are in Western society. Reducing the stigma surrounding AVH would likely impact on how the person reacts emotionally and behaviorally to them. Furthermore, we should now be intervening to reduce the incidence and impact of childhood adversity, which is a key risk factor for AVH and other psychotic symptoms.^99^

### What Methodological Issues Should be Addressed in Future Studies?

Studies of individuals with AVH without need for care often have varying inclusion and exclusion criteria. Including a diagnostic interview would be helpful, together with measures of other subclinical symptoms (eg, anxiety, depression, and delusional ideation), as their presence may have an impact on AVH (see supplementary appendix). It would also be very helpful to learn more about the type, significance, and content of the voices experienced. More sensitive assessment tools (especially self-report) are needed to identify those with AVH who might be at risk of transitioning to a clinical disorder, and we recommended that studies follow up screening measures with detailed interviews. We need replications of findings in other samples of individuals with AVH without need for care because most of our knowledge about nonclinical, frequent AVH is based on the sample recruited by Iris Sommer’s group. Combining longitudinal and cross-sectional methodologies may also be productive.

Overall, greater methodologial rigor is needed to advance our understanding of AVH in persons with and without a need for care. This involves using similar inclusion criteria and assessments of participants, and minimizing confounding variables, in order to improve the comparability of results across different studies. This action point will be taken forward by the Consortium.

## Supplementary Material

Supplementary material is available at http://schizophreniabulletin.oxfordjournals.org.

## Funding

Regione Sardegna, Italy (PRR-MAB-A2011-19251 to S.S.); European Research Council advanced grant to Kenneth Hugdahl (ERC, Voice 249516 to K.K.); Australian National Health and Medical Research Council (APP1046216 to J.S.); Wellcome Trust (WT098455 to C.F., B.A.-D., S.Mc.-J.).

## Supplementary Material

Supplementary Data
