# Supplementary material for: Auditory Verbal Hallucinations in Persons With and Without a Need for Care
Source: Schizophr Bull. 2014 Jun 13;40(Suppl 4):S255–64. doi: 10.1093/schbul/sbu005 (PMC4141313; doi:10.1093/schbul/sbu005)
Supplement: Supplementary Data [file supp_40_Suppl-4_S255__index.html]

Supplementary Data 

# Auditory Verbal Hallucinations in Persons With and Without a Need for Care

## Supplementary Data

Data files

**Files in this Data Supplement:**

- Supplementary Data - Supplementary Data
