# Supplementary material for: Auditory Verbal Hallucinations in Persons With and Without a Need for Care
Source: Schizophr Bull. 2014 Jun 13;40(Suppl 4):S255–64. doi: 10.1093/schbul/sbu005 (PMC4141313; doi:10.1093/schbul/sbu005)
Supplement: Supplementary Data [file supp_sbu005_APPENDIX_R1.pdf]

APPENDIX

Assessment of hallucinations

A large number of assessment instruments assess AVH/hallucinations in non-clinical samples, with three categories briefly reviewed here: self-report; detailed instruments; and those that evaluate beliefs, attitudes and coping with AVH (see Table 1 for summaries of some of these). Some scales can be distinguished as to whether they were designed to be used for clinical or non-clinical populations. Although instruments used for clinical populations could also be used for non-clinical populations, scales designed for clinical populations are usually very detailed, and their use is only appropriate in those non-clinical persons who experience articulated hallucinations. There is no reason why instruments originally designed for non-clinical populations (e.g. self-report measures) cannot also be used for exploratory purposes in clinical populations.

(A) Self-report measures

Brief self-report measures are suitable for screening large samples or use in experimental studies when interview is not feasible. Key issues for self-report measures include the importance of defining a timeline (e.g. lifetime, within the last month) and excluding hallucinations associated with drugs, fatigue, etc. It is preferable to avoid dichotomous response scales and to use dimensional ones (Likert scales) both for statistical purposes and to avoid defensive responding. Also, it is preferable to assess several hallucination modalities, not just auditory, and to be able to extract specific factor scores (e.g. an AVH score). Having participants rate supplementary dimensions (e.g. degree of distress, conviction, intrusiveness, frequency) is additionally informative and increases sensitivity of the measure (e.g. only include those with hallucinations that are also distressing). Self-report measures are particularly suitable in experimental contexts where just one independent variable (e.g. a total score or a sub score/factor score based on specific target items) is needed to group participants (e.g. high versus low hallucination-proneness). Many self-report measures contain items not only related to overt hallucinations, but also to other related experiences (e.g. vivid and intrusive thoughts, vivid daydreams, felt-realness), making the instrument less “transparent”. Finally, a disadvantage of these measures is that limited detail is recorded about the hallucinations.This may be offset by using a self-report scale as a screening measure and then, for those who report hallucinations, administering more detailed instruments.

(B) Detailed measures

Several more detailed measures exist, which may be semi-structured or structured interviews. In general, they assess characteristics such as frequency, duration, location, loudness, localisation, beliefs of origin, emotional aspects (amount and degree of negative content, emotional state during, associated emotions), the disruption to life caused by voices, controllability, clarity, form and content (e.g., linguistic complexity, repeated contents, commands), and psychosocial aspects (e.g., triggers, coping strategies used, role of medication).

(C) Other measures

Others measures focus specifically on beliefs, attitudes and coping with AVH. These are useful when one requires information that extends beyond the perceptual characteristics of hallucinations.

Finally, in general, it is useful to consider including a diagnostic interview to determine the presence or absence of diagnoses, plus measures of other co-existing subclinical symptoms (e.g. anxiety, depression, delusional ideation, OCD) as their presence may have an impact on hallucinations.

INSERT TABLE 1 HERE

	**What is assessed?**	**Category**	**Format**	**Main reference**
Launay Slade Hallucinations Scale (LSHS)	Halls + related experiences	A	Self-report	Launay, G. & Slade, P. (1981). The measurement of hallucinatory predisposition in male and female prisoners. Personality and Individual Differences, 2, 221–234
LSHS Revised version	Halls + related experiences	A	Self-report	Morrison, A.P. et al. (2000). Cognitive factors in predisposition to auditory and visual hallucinations. British Journal of Clinical Psychology, 39, 67-78
LSHS Extended version	Halls + related experiences	A	Self-report	Larøi, F., & Van der Linden, M. (2005). Normal subjects' reports of hallucinatory experiences. Canadian Journal of Behavioural Science, 37, 33-43
Cardiff Anomalous Perceptions Scale	Halls + related experiences	A	Self-report	Bell, V. et al. (2006). The Cardiff Anomalous Perceptions Scale (CAPS): A new validated measure of anomalous perceptual experience. Schizophrenia Bulletin, 32, 366-377
Community Assessment of Psychic Experiences	Halls + other symptoms	A	Self-report	Konings, M. et al. (2006). Validity and reliability of the CAPE: a self-report instrument for the measurement of psychotic experiences in the general population. Acta Psychiatrica Scandinavica, 114, 55–61
Structured Interview for Assessing Perceptual Anomalies	Halls + related experiences	B	Structured interview	Bunney, W. et al. (1999). Structured Interview for Assessing Perceptual Anomalies (SIAPA). Schizophrenia Bulletin, 25, 577-592
Psychotic Symptom Rating Scales	AVH	B	Semi-structured interview	Haddock, G. et al., (1999) Scales to measure dimensions of hallucinations and delusions: the psychotic symptom rating scales (PSYRATS). Psychological Medicine, 29, 879-889
The Mental Health Research Institute Unusual Perception Schedule	AVH	B	Semi-structured interview	Carter, D. M. et al. (1995). The development and reliability of the Mental Health Research Institute Perceptions Schedule (MUPS): An instrument to record auditory hallucinatory experience. Schizophrenia Research, 16, 157-165
Auditory Vocal Hallucination Rating Scale	AVH	B	Structured interview	Bartels-Velthuis, A.A. et al. (2012). Consistency and reliability of the Auditory Vocal Hallucination Rating Scale (AVHRS). Epidemiology and Psychiatric Sciences, 21, 305-310
Interview with a person who hears voices	AVH	B	Semi-structured interview	Romme, M. A., & Escher, A. D. (1989). Hearing voices. Schizophrenia Bulletin, 15, 209–216
Interpretations of Voices Inventory	Beliefs about AVH	C	Self-report	Morrison, A. P. et al. (2002). Cognitive and emotional predictors of predisposition to hallucinations in non-patients. British Journal of Clinical Psychology, 41, 259-270
Revised Beliefs About Voices Questionnaire	Beliefs about AVH	C	Self-report	Chadwick, P. et al. (2000) The revised beliefs about voices questionnaire (BAVQ-R). Br J Psychiatry 177, 229-232
The Voice and You	Relationship between hearer and AVH	C	Semi-structured interview	Hayward, M. et al. (2008) The Voice and You: Development and psychometric evaluation of a measure of relationships with voices. Clinical Psychology and Psychotherapy, 15, 45-52
Voices Acceptance and Action Scale	Acceptance-based attitudes and actions in relation to AVH and command hallucinations	C	Self-report	Shawyer, F. et al. (2007). The Voices Acceptance and Action Scale (VAAS): Pilot data. Journal of Clinical Psychology, 63, 593-606
Maastricht Assessment of Coping Strategies	Coping strategies of various psychotic symptoms including halls	C	Semi-structured interview	Bak, M. et al. (2001). Maastricht Assessment of Coping Strategies (MACS-I): A brief instrument to assess coping with psychotic symptoms. Acta Psychiatrica Scandinavica, 103, 453-459.

Table 1: Summary of some central hallucination measures that may be used when assessing AVH in individuals without need for care
