# Supplementary material for: Auditory Verbal Hallucinations in Persons With and Without a Need for Care
Source: Schizophr Bull. 2014 Jun 13;40(Suppl 4):S255–64. doi: 10.1093/schbul/sbu005 (PMC4141313; doi:10.1093/schbul/sbu005)
Supplement: Supplementary Data [file supp_sbu005_APPENDIX_R1.doc]

**APPENDIX**

**Assessment of hallucinations**

A large number of assessment instruments assess AVH/hallucinations in non-clinical samples, with three categories briefly reviewed here: self-report; detailed instruments; and those that evaluate beliefs, attitudes and coping with AVH (see Table 1 for summaries of some of these). Some scales can be distinguished as to whether they were designed to be used for clinical or non-clinical populations. Although instruments used for clinical populations could also be used for non-clinical populations, scales designed for clinical populations are usually very detailed, and their use is only appropriate in those non-clinical persons who experience articulated hallucinations. There is no reason why instruments originally designed for non-clinical populations (e.g. self-report measures) cannot also be used for exploratory purposes in clinical populations.

*(A) Self-report measures*

Brief self-report measures are suitable for screening large samples or use in experimental studies when interview is not feasible. Key issues for self-report measures include the importance of defining a timeline (e.g. lifetime, within the last month) and excluding hallucinations associated with drugs, fatigue, etc. It is preferable to avoid dichotomous response scales and to use dimensional ones (Likert scales) both for statistical purposes and to avoid defensive responding. Also, it is preferable to assess several hallucination modalities, not just auditory, and to be able to extract specific factor scores (e.g. an AVH score). Having participants rate supplementary dimensions (e.g. degree of distress, conviction, intrusiveness, frequency) is additionally informative and increases sensitivity of the measure (e.g. only include those with hallucinations that are also distressing). Self-report measures are particularly suitable in experimental contexts where just one independent variable (e.g. a total score or a sub score/factor score based on specific target items) is needed to group participants (e.g. high versus low hallucination-proneness). Many self-report measures contain items not only related to overt hallucinations, but also to other related experiences (e.g. vivid and intrusive thoughts, vivid daydreams, felt-realness), making the instrument less “transparent”. Finally, a disadvantage of these measures is that limited detail is recorded about the hallucinations.This may be offset by using a self-report scale as a screening measure and then, for those who report hallucinations, administering more detailed instruments.

*(B) Detailed measures*

Several more detailed measures exist, which may be semi-structured or structured interviews. In general, they assess characteristics such as frequency, duration, location, loudness, localisation, beliefs of origin, emotional aspects (amount and degree of negative content, emotional state during, associated emotions), the disruption to life caused by voices, controllability, clarity, form and content (e.g., linguistic complexity, repeated contents, commands), and psychosocial aspects (e.g., triggers, coping strategies used, role of medication).

*(C) Other measures*

Others measures focus specifically on beliefs, attitudes and coping with AVH. These are useful when one requires information that extends beyond the perceptual characteristics of hallucinations.

Finally, in general, it is useful to consider including a diagnostic interview to determine the presence or absence of diagnoses, plus measures of other co-existing subclinical symptoms (e.g. anxiety, depression, delusional ideation, OCD) as their presence may have an impact on hallucinations.

INSERT TABLE 1 HERE
